# Supplementary material for: ProteinShader: illustrative rendering of macromolecules
Source: BMC Struct Biol. 2009 Mar 30;9:19. doi: 10.1186/1472-6807-9-19 (PMC2672931; doi:10.1186/1472-6807-9-19)
Supplement: Additional file 1 — ProteinShader program without source code. This compressed file contains the complete ProteinShader program including associated libraries, but no source code. A README.txt file gives an overview of the ProteinShader distribution, and the index.html file in the help subdirectory has directions on getting started with the program as well as a set of tutorials. [file 1472-6807-9-19-S1.zip › ProteinShader-beta-0_9_4-binary/help/api/org/proteinshader/gui/listeners/controlpanel/RadioPanelListenerFactory.html]

RadioPanelListenerFactory (ProteinShader API)


|  |  |  |
| --- | --- | --- |
| **Overview** \| **Package** \| **Class** \| **Use** \| **Tree** \| **Deprecated** \| **Index** \| **Help** | |  |
| **PREV CLASS**   **NEXT CLASS** | **FRAMES**    **NO FRAMES**     **All Classes** |  |
| SUMMARY: NESTED \| FIELD \| CONSTR \| METHOD | DETAIL: FIELD \| CONSTR \| METHOD |  |


---


## org.proteinshader.gui.listeners.controlpanel Class RadioPanelListenerFactory

```
java.lang.Object
  org.proteinshader.gui.listeners.controlpanel.RadioPanelListenerFactory
```

---

``` public class RadioPanelListenerFactory extends Object ```

Creates the listener for the RadioPanel.

---

**Constructor Summary**

`RadioPanelListenerFactory(RadioPanel radioPanel)`
  
          Constructs a RadioPanelListenerFactory.


| **Method Summary** | |
| --- | --- |
| `ActionListener` | `createMenuActionListener()`             Creates an action listener for the combo box menus in the RadioPanel. |
| `ActionListener` | `createRadioButtonsActionListener()`             Creates an action listener for the radio buttons. |

**Methods inherited from class java.lang.Object**

`clone, equals, finalize, getClass, hashCode, notify, notifyAll, toString, wait, wait, wait`

**Constructor Detail**

### RadioPanelListenerFactory

```
public RadioPanelListenerFactory(RadioPanel radioPanel)
```

:   Constructs a RadioPanelListenerFactory.

    **Parameters:**: `radioPanel` - the RadioPanel that this class creates listeners for.


**Method Detail**

### createRadioButtonsActionListener

```
public ActionListener createRadioButtonsActionListener()
```

:   Creates an action listener for the radio buttons. When the action
    listener is called, it will call setActiveRadioButton() on the
    RadioPanel. When a client of the RadioPanel needs to make a
    modification, it can find out which radio button is currently
    active by calling getActiveRadioButton().

    :   **Returns:**: The action listener.

---


### createMenuActionListener

```
public ActionListener createMenuActionListener()
```

:   Creates an action listener for the combo box menus in the
    RadioPanel. If a user has made a selection from the menu, he/she
    will likely expect the corresponding radio button to be marked to
    indicate that the menu is now the current choice that will be used
    when the "Apply" or "Default" button is pressed. Therefore, the
    this action listener will update the RadioPanel with the current
    choice of menu to use.

    :   **Returns:**: The action listener.


---


|  |  |  |
| --- | --- | --- |
| **Overview** \| **Package** \| **Class** \| **Use** \| **Tree** \| **Deprecated** \| **Index** \| **Help** | |  |
| **PREV CLASS**   **NEXT CLASS** | **FRAMES**    **NO FRAMES**     **All Classes** |  |
| SUMMARY: NESTED \| FIELD \| CONSTR \| METHOD | DETAIL: FIELD \| CONSTR \| METHOD |  |


---

# *Copyright © 2007-2008*
